# Infectious spondylodiscitis and kyphosis correction in an infant: a case report

**DOI:** 10.1186/s13052-021-01106-4

**Published:** 2021-07-05

**Authors:** Sara Romano, Francesca Vittoria, Elisabetta Cattaruzzi, Egidio Barbi, Marco Carbone

**Affiliations:** 1 Department of Medicine, Surgery, and Health Sciences -University of Trieste, Trieste, Italy; 2 Institute for Maternal and Child Health - IRCCS “Burlo Garofolo”,, Trieste, Italy

**Keywords:** Spondylodiscitis, Infant, Bony destruction, Kyphosis

## Abstract

**Background:**

Neonatal infectious spondylodiscitis is a rare bony infection with atypical clinical presentation and non-specific systemic symptoms. Diagnosis and treatment are often delayed resulting in vertebral destruction and severe complications. We retrospectively reviewed the case of an infant with infectious spondylodiscitis resulting in T12 body destruction and marked angular kyphosis.

**Case-report:**

A 4-week-old infant developed an infectious spondylodiscitis resulting in destruction of the T12 vertebral body and involvement of disc between T12 and L1. At 6 months of age, X-ray showed a marked thoracolumbar angular kyphosis above 50 Cobb degrees. Therefore, the patient underwent single time surgery with double anterior and posterior approach. At 9 years follow up, clinical and radiological findings show a stable correction with good aesthetic appearance.

**Conclusion:**

Neonatal spondylodiscitis could lead to marked kyphosis similar to the congenital one. Since treatment with casts and tutors is often inefficacious, prompt surgery should be considered. The double anterior and posterior approach is the best option in this condition.

## Introduction

Primary pyogenic spinal infections are uncommon entities in children. The estimated incidence of discitis and spondylodiscitis is 1–2 cases per year per 32,500 pediatric hospital evaluations [1], representing approximately 3% of all the cases of osteoarticular infections [2]. The age distribution of pediatric spondylodiscitis is triphasic: the first peak occurs in early childhood (79%), between the age of 6 months and 4 years, a smaller later peak in the juvenile and adolescent group (20%), and only exceptional infections in children aged under 6 months (1%) [3]. The lumbar region is the spine level predominately involved, and the most common causative organism is *Staphylococcus aureus* [4]. Unlike older children, neonates and infants with spondylodiscitis are often systemically ill with the involvement of multiple infectious foci. Therefore, early diagnosis and treatment are crucial as the vertebrae can be severely damaged or entirely destroyed [5, 6].

We present an infant who developed hematogenous spondylodiscitis resulting in vertebral body destruction and marked angular kyphosis. The deformity was surgically treated, leading to a stable overtime correction.

## Case presentation

**Fig. 1:**
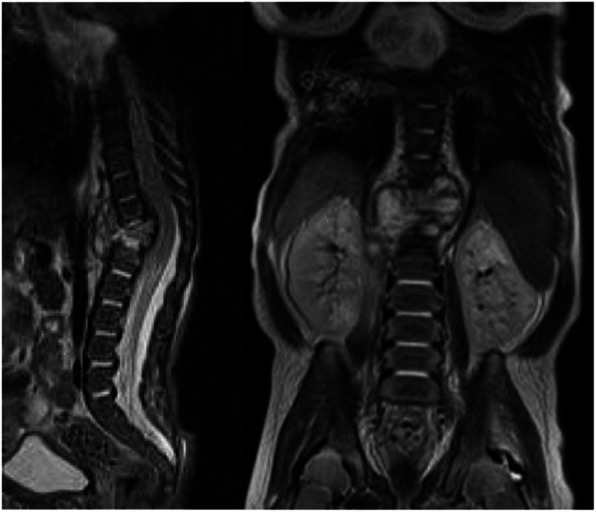
MRI at 5 weeks of age showing the destruction of the T12 vertebral body with involvement of disc between T12 and L1

A 4-week-old boy presented to the emergency department with a history of hyporeactivity, feeding difficulties and failure to thrive. On examination, he was pale, hyporeactive with dystrophic appearance. After 10 days of hospitalization, he developed low-grade fever (37.5 °C maximum temperature) and a tender spinal tumefaction in the thoracolumbar region. The lower extremities did not present limitations of movement, asymmetries, or pain under passive mobilization. The whole-body magnetic resonance imaging (MRI) showed the destruction of the T12 vertebral body with the involvement of disc between T12 and L1 (Fig.1). The laboratory tests showed a white blood cell count of 18.3 × 10^9^ per liter, with a differential of 54.9% neutrophils and 28.0% lymphocytes. The C-reactive protein was 1.19 mg/dL (normal value < 0.5 mg/dL). A vertebral body osteomyelitis was suspected and an empiric antibiotic therapy with tobramycin, vancomycin and amphotericin was started. Blood cultures were positive for *Staphylococcus aureus* and intravenous vancomycin only was continued for total 6 weeks. A thorough physical reevaluation could not highlight a primary focus of Staphylococcus infection leading to the spondylodiscitis.

**Fig. 2:**
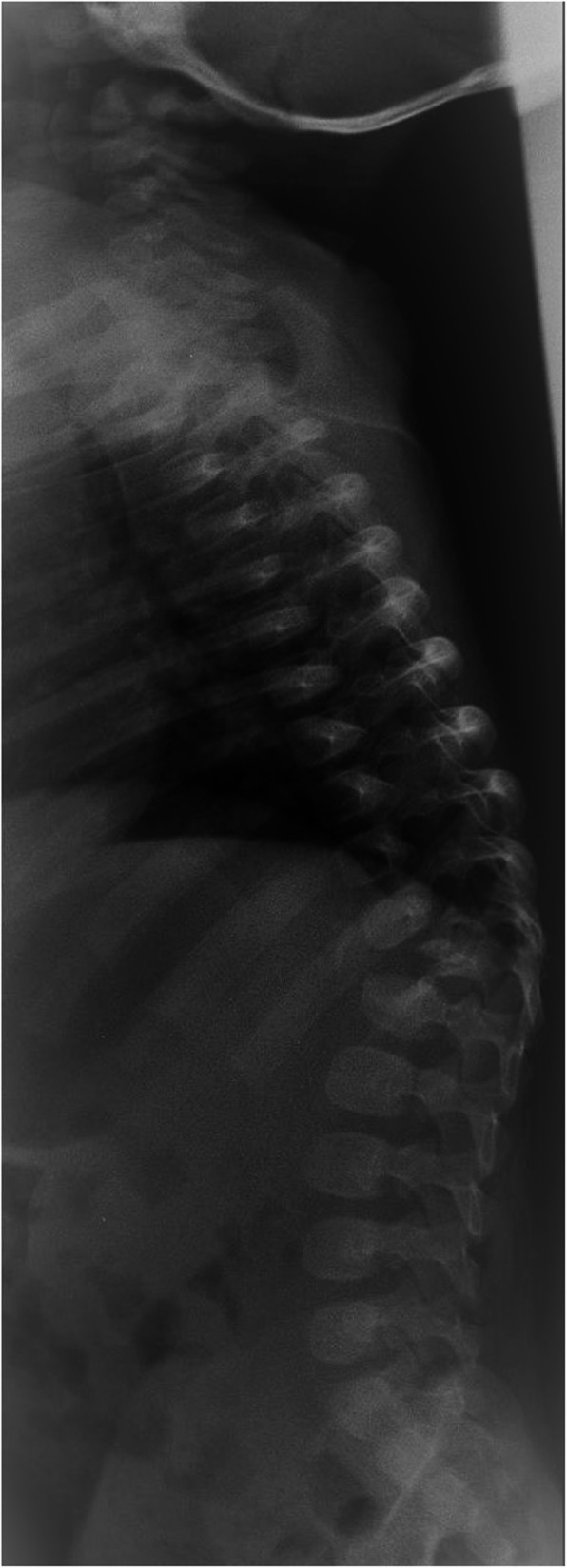
Lateral radiograph at 6 months of age shows the almost complete destruction of T12 vertebral body, resulting in angular kyphosis above 50°

**Fig. 3:**
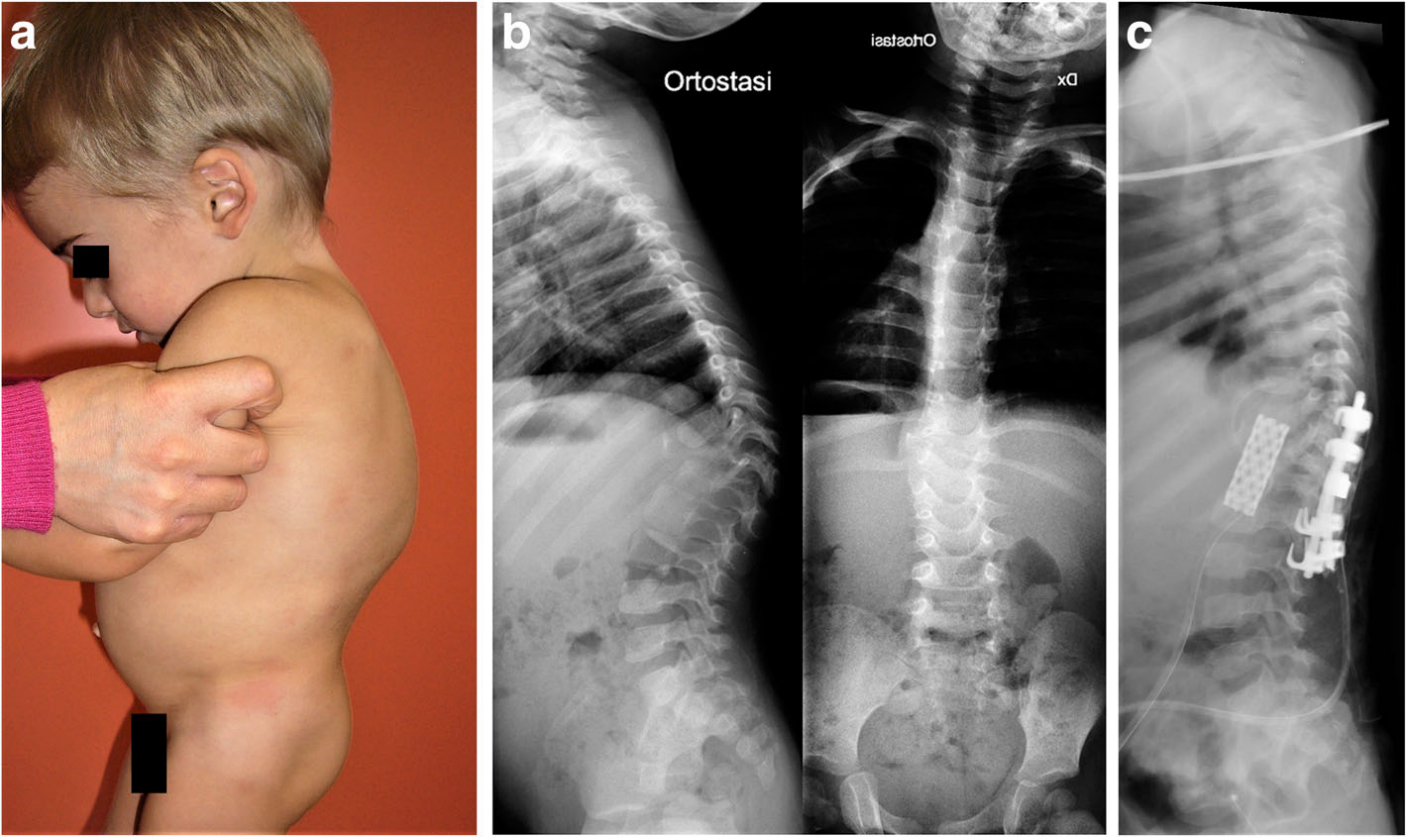
Pre-operatory clinical aspect (**a**), plain radiographs at 2 years of age showing the persistent and progressive kyphosis (**b**) and post-operatory standing lateral radiograph showing the double approach with posterior hooks and anterior bank bone graft titanium cage from T11 to L2 (**c**)

At the age of 6 months, X-ray showed almost complete destruction of T12 body and L1 body partially, with evidence of thoracolumbar angular kyphosis above 50 Cobb degrees. Kyphosis worsened in the following months despite the Milwaukee brace use (Fig. 2). Therefore, at the age of 2 years, he underwent circumferential vertebral fusion from T11 to L2: the hooks were posteriorly instrumented, while a titanium cage of bank bone graft was positioned anteriorly, through left retroperitoneal access (Fig. 3).

**Fig. 4:**
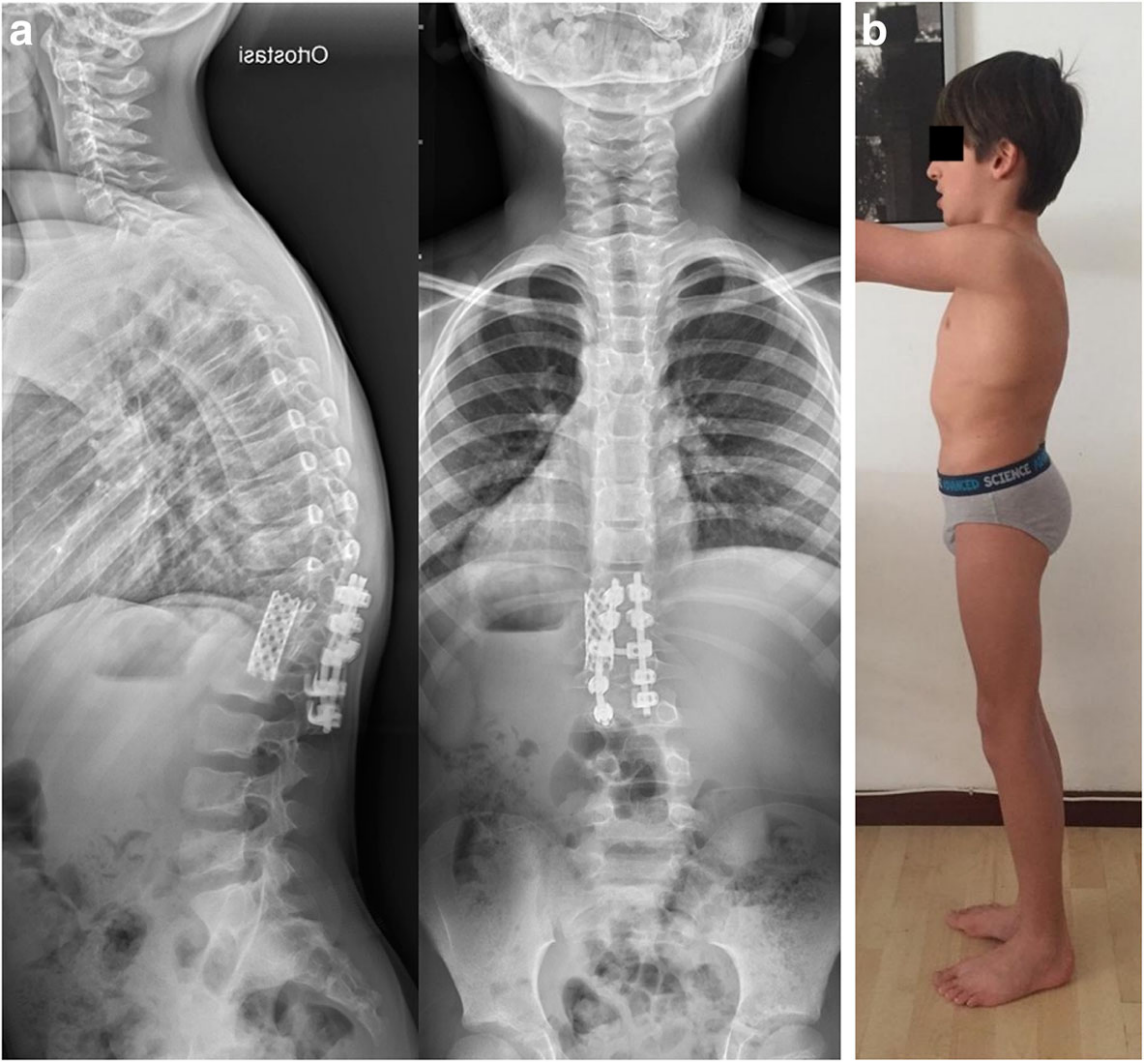
Standing radiographs (**a**) and clinical aspect (**b**) showing the permanent deformity correction at 9 years of age

At present, 9 years after the operation, the patient leads a normal life, no neurological deficiencies were detected, and X-ray shows a stable correction with spine sagittal curves within the physiological limits (Fig. 4).

## Discussion

Neonatal infectious spondylodiscitis accounts for 2 to 4% of bony infections in neonates [7]. Risk factors include prematurity with low birth weight, catheterization of the umbilical vessel, and other invasive procedures [8].

*Staphylococcus aureus* is the most prevalent cause in approximately 80 to 90% of cases. Other agents, less commonly identified, are *coagulase-negative Staphylococcus*, *α-hemolytic Streptococcus*, *Streptococcus pneumoniae*, *Escherichia coli* and *Salmonella* spp. [4]. In most patients, pathogens reach the spine by hematogenous route, involving first the disc and subsequently the adjacent vertebral endplates through supply vessels that generally persist until the seventh year of life^4^. Due to the atypical presentation, diagnosis is usually delayed leading to extensive vertebral destruction and subsequent deformation [5, 6]. Magnetic resonance is the first-choice imaging, allowing the delineation of spinal cord compression, bony destruction, and abscess extension [9]. Invasive investigations, such as biopsy or aspirations, should be conducted on children who fail to improve with antibiotic treatment or when the presence of atypical organisms is suspected [5].

The fusion of kyphosis of this type requires the execution of a posterior and anterior arthrodesis to obtain a result that remains stable over time. Considering the patient size, an autologous bone grafting was not feasible. In the anterior surgery, it is advisable to use support with good mechanical strength; although a strut graft could be taken into account, we preferred using a titanium cervical cage filled with bank bone grafts. This rigid structure showed moderate subsidence but was overall stable in maintaining the correction obtained. This stability was obtained, limiting the fusion to five vertebrae, with little consequences on the trunk motility and good aesthetic appearance.

## Conclusion

Neonatal spondylodiscitis could lead to vertebral body destruction with resulting angular kyphosis. It is often similar to congenital forms in terms of the type of deformity, radiological aspect, and indications for treatment [10, 11]. Treatment with plaster or orthopedic brace is doomed to failure. Surgery must be performed at an early stage, as soon as worsening occurs to avoid the onset of more severe deformities, possible neurological damage, and higher surgical risks.

## Data Availability

No supporting data are available.
